# Complete Genome Sequences of Mycobacterium bovis Strains Affiliated with Bovine Tuberculosis Outbreaks in Canada in 2016 and 2018

**DOI:** 10.1128/mra.01213-22

**Published:** 2023-02-14

**Authors:** Olga Andrievskaia, Amalia Garceac, Dara Lloyd, Mirjana Savic, Marc-Olivier Duceppe

**Affiliations:** a Canadian Food Inspection Agency, Ottawa Laboratory Fallowfield, Ottawa, Ontario, Canada; University of Rochester School of Medicine and Dentistry

## Abstract

Mycobacterium bovis is the primary causative agent of bovine tuberculosis, a zoonotic infectious disease that presents a risk to public health, livestock, and wildlife. Here, we report complete genome sequences of two Mycobacterium bovis strains affiliated with bovine tuberculosis outbreaks in Canadian cattle farms in 2016 and 2018.

## ANNOUNCEMENT

Bovine tuberculosis (bTb) is an infectious zoonotic disease caused by Mycobacterium bovis and can present a risk to public health, the livestock industry, and wild animal species (1). Bovine tuberculosis is largely considered to be eliminated from Canadian livestock (2), with only two sporadic localized outbreaks detected in cattle farms over the past decade. Whole-genome sequencing (WGS) was performed for M. bovis strains affiliated with these outbreaks to provide references for epidemiological investigations and comparisons with M. bovis strains observed historically in Canada (3). The M. bovis strains 2016/0386 and 2018/0565 were isolated from mycobacteriosis-compatible granulomas collected from cattle in the provinces of Alberta in 2016 and British Columbia in 2018, respectively. M. bovis culture isolation and identification were performed at the National Reference Laboratory for Bovine Tuberculosis, Canadian Food Inspection Agency, according to standard microbiological methods (4). M. bovis isolates were stored in Proskauer-Beck medium supplemented with 5% horse serum at −80°C. For WGS, M. bovis isolates were grown on Middlebrook 7H11 agar for 4 weeks. Genomic DNA was purified using a combination of mechanical cell lysis with 0.5-mm ceramic beads (OMNI International, USA) and the MasterPure Gram-positive DNA purification kit (Lucigen, USA). Illumina sequencing libraries were prepared using Nextera XT DNA Library Preparation kit (Illumina Inc., USA) and sequenced on a MiSeq sequencer (Illumina Inc.) using a MiSeq reagent kit v2 to generate 2 × 250-bp paired-end sequences. For Nanopore sequencing, DNA samples were treated with a Short Read Eliminator XS kit (Circulomics, USA). Nanopore libraries were prepared using the 1D Native barcoding genomic DNA protocol with EXP-NBD104 and SQK-LSK109 kits (Oxford Nanopore Technologies, UK) without shearing and sequenced using FLO-MIN111 flow cells on a MinION Mk1B device (Oxford Nanopore Technologies). The Nanopore basecalling was performed using Super Accuracy mode in Guppy v5.0.11, trimmed using Porechop v0.2.3, and filtered using Filtlong v0.2.1 (5). Long read assembly was performed with Trycycler (6) using Flye v2.7 (7), necat v0.0.1 (8), Canu v2.2 (9), and Shasta v0.8.0 (10). Reads were split in six subsamples. Circularized consensus sequences were corrected using Medaka v1.4.4 and polished with Illumina MiSeq reads using a combination of NextPolish v1.4.0 (11), ntEdit v1.3.5 (12), and Polypolish v0.5.0 (13) after trimming/filtering with fastp v0.23.2 (14). The sequencing coverage depth was determined using Minimap2 v2.17 (15) and SAMtools v1.7 (16) for long reads and BWA v0.7.17 and SAMtools for short reads. Default parameters of the above bioinformatics software were used unless otherwise specified (https://github.com/OLF-Bioinformatics/nanopore/blob/master/run_trycycler.sh; https://github.com/OLF-Bioinformatics/nanopore/blob/master/short_read_polish.sh). Gene prediction and annotation were done using NCBI Prokaryotic Genome Annotation Pipeline 2022-04-14.build6021 (17).

Assembly metrics and genomic characteristics are summarized in Table 1. The complete genomes of M. bovis strains 2016/0386 and 2018/0565 contain single chromosomes with a G+C content of 65.6%, 45 tRNAs, and 2 clustered regularly interspaced short palindromic repeats (CRISPRs). FastANI (v1.33) (18) whole-genome comparison of strains 2016/0386 and 2018/0565 with a reference M. bovis strain AF2122/97 (19) generated an average nucleotide identity of 99.98%.

**TABLE 1:** Characteristics of complete genome sequences of two Mycobacterium bovis strains affiliated with bTb outbreaks in Canada in 2016 and 2018

M. bovis strain information			No. of reads by platform		Nanopore read *N*_50_ (bp)	Coverage (×)	Chromosome size (bp)	Predicted no. of CDSs^*a*^	Predicted no. of genes	Accession no. by database		
										GenBank no.	SRA Nanopore	SRA Illumina
Name	Origin	Yr	Illumina	Nanopore								
2016/0386	Alberta	2016	3,840,394	145,106	7,731	394	4,347,959	4,025	4,076	CP112997	SRR22314236	SRR22314237
2018/0565	British Columbia	2018	14,581,052	61,840	5,842	576	4,350,529	4,022	4,073	CP112996	SRR22314234	SRR22314235

a CDSs, coding DNA sequences.

### Data availability.

The assembled closed genome sequences of M. bovis strains 2016/0386 and 2018/0565 and raw reads are deposited in the NCBI GenBank under the BioProject number PRJNA902023. The accession numbers are listed in Table 1. The versions described in this paper are the first versions.
